# Rhinology, Laryngology and Otology

**Published:** 1890-09

**Authors:** Frank Hamilton Potter


### Progress in Medical Science.

### RHINOLOGY, LARYNGOLOGY, AND OTOLOGY.

By FRANK HAMILTON POTTER, M. D.

### ANESTHESIA IN SMALL SURGICAL OPERATIONS.

Dr. Ad. Barth, of Berlin, (*Archives of Otology*, April-July, 1890,) contributes some observations on Cocainization, Electrical Cataphoresis, and Hypnotism and Suggestion, as employed for the purpose of producing anesthesia in small surgical operations. In regard to cocaine, he says that while it cannot be recommended for continuous local treatment in chronic inflammation of mucous membranes, yet it retains its permanent value for local anesthesia, for reducing a swelling, for diagnostic and therapeutic purposes, especially in the nose, and eventually as a palliative measure in acute irritation and inflammation. Electrical cataphoresis is the method of employing cocaine in operations upon the ear. The ordinary method of its application proving unsatisfactory, Wagner suggested it being used in connection with the electrical current. Dr. Barth proceeds as follows : An eight to ten per cent. solution of cocaine connected with the positive pole was permitted for fifteen or twenty minutes to act upon the part to be anesthetized. The negative pole was placed upon the nape of the neck. The current was produced by four to ten Glauert's (Spamer's) elements, according to how recently the apparatus was filled or to the sensibility of the patient. For application to the ear the author simply rolled some cotton around the wire point, which is screwed for other purposes into the handle of the positive electrode, using it like a cotton carrier, and introducing it into the external auditory meatus. The external portion of the meatus must be protected by cotton against the direct contact of the wire with the skin. After considerable experience with this method, he concludes that in many cases sensibility was entirely abolished, and in most it was extremely diminished. Any tendency to vertigo can be avoided by the simultaneous electrization of both sides.

The rest of the article is devoted to the consideration of the value of hypnosis, both as an anesthetic for operations upon the upper air-passages, and as a therapeutic measure in some of the diseases seen there. A few cases are given in illustration. While hypnotism is considered a valuable aid, it ought to be used by those only who are experts in regard to it. Dr. Barth has never seen injurious effects from its employment, and thinks that when they occur the fault is not to be found in hypnotism, but in the hypnotizer. One practical point may be given in his own words :

> Since we never know beforehand how deep the hypnosis will become, and, principally, whether we will succeed in producing thereby anesthesia, I have *always simultaneously used local anesthesia in operations, leaving to the hypnosis only the removal of the undesirable action of the psyche.*

Is it not pertinent to inquire how Dr. Barth can estimate the anesthetic power of hypnosis if he always uses simultaneously with it local anesthesia ?

In conclusion, he quotes with approval the words of Forel :

> The application of the entirely safe suggestion (I do not mention the dangers incident to its unskilful, criminal or improper use) will no doubt gradually occupy the position it deserves in therapeutics ; its indications should be rendered more precise, and freed from exaggerations, and the correct manipulation should be learned.

And he believes that hypnosis will secure for itself a permanent place in the diagnostic and therapeutic management of hysterical deafness, subjective noises, hyperesthesia, and like conditions, as well as in small operations.

### RUPTURES OF THE MEMBRANA TYMPANI.

Leopold Treitel, of Breslau (*Ibid*), concludes a paper on Ruptures of the Membrana Tympani, with especial reference to their Forensic Importance, with the following remarks concerning the treatment : The chief rule is to do no harm. A trivial injury of the membranum tympani, unskilfully treated, may become the starting point of a chronic middle-ear suppuration, with all its disagreeable consequences. The best treatment is simply to close the meatus with a wad of absorbent cotton, and to avoid all use of the syringe. There are plenty of physicians who still use the syringe in these cases, but it should not be used unless suppuration ensued, and even then with great caution. Furthermore, the forcing of air through the tubes by the physician or the patient ought to be most carefully avoided. And, on the whole, if there are no further complications, the patient can attend to his affairs as usual.

### UNDECIDED QUESTIONS CONCERNING ATROPHIC RHINITIS.

Lennox Browne, of London, (*Journal of Laryngology and Rhinology*, July, 1890,) in a paper entitled, On the Classification of Intra-nasal and Naso-Pharyngeal Diseases, reviews some of the questions as yet undecided relating to diseases as seen in these localities. In regard to atrophic rhinitis, we must consider whether it is ever a sequel to hypertrophic rhinitis, or, as asserted by Bosworth, an entirely separate disease. Are there not varieties of atrophic rhinitis ? What relation do the constitutional dyscrasiæ, such as anemia, struma, syphilis, etc., bear to the disease ? What influence has alcohol in its production ? In what degree is it influenced by disorder of the portal circulation, and what is the importance as an etiological factor of sexual irritation, delayed menstruation, menorrhagia, and other uterine floodings ? Is there any constancy or unity of bacterial association ? Is it ever the direct sequence of an exanthem, or of unsanitary surroundings ? Further statistics and facts are also required on the peculiar physiognomy of the subjects of atrophic rhinitis. Is there always an upturned and abnormally patent nostril ? Lastly, is it ever curable ? Answers must be sought to these questions before we can advance our knowledge of atrophic rhinitis.

### ON SOME COMPLICATIONS FOLLOWING THE REMOVAL OF ADENOID GROWTHS FROM THE NASO-PHARYNX.

Dr. A. Cartaz (*Revue de Laryngologie, D' Otologie et de Rhinologie*, for July 15, 1890,) states while the hemorrhage following the removal of adenoid growths from the pharyngeal vault is generally easily controlled, yet, sometimes, it is quite severe. He quotes four cases reported by D. Bryson Delavan, of New York, in which the bleeding was so severe as to cause anxiety. He also reports two cases as occurring in his own practice. Besides these he has collected a few other cases — one by Segond, two by Ruault, one by Gellé, and one by W. Scott Renner ; the latter was reported in the BUFFALO MEDICAL AND SURGICAL JOURNAL for April, 1890. The author holds that these hemorrhages do not result from the wounding of an important vessel — they are rather

"hemorrhagies en nappe," and are more liable to occur when the growths are fibrous in character. Severe bleeding is also more likely to occur when the parts are in a state of congestion from inflammatory attacks. The treatment consists of astringent irrigations as hot as can be tolerated, and the plugging of the naso-pharynx. The plug must be large enough to pass into the posterior nares.

There has also been observed after these operations septic fever, facial erysipelas, tonsillitis and inflammations of the middle ear. These are all rare, and when they occur are usually of a mild type. They disappear in a few days under appropriate treatment. Cartaz agrees with Schaeffer in condemning inflation of the Eustachian tubes immediately after operation. It is apt to project blood and the débris of the vegetations into the tubes and thus excite serious inflammations. On the other hand, irrigations of the pharyngeal vault has never in his hands led to any trouble. He counsels the employment of antisepsis, especially as to the instruments, in order to prevent the development of any septic trouble. He believes, also, that the thorough washing out of the nose and naso-pharynx for a few days previous to the operation with a five per cent. solution of boric acid, to be of benefit.

### SALOL IN ACUTE TONSILLITIS AND PHARYNGITIS.

Jonathan Wright, of New York, (*American Journal of the Medical Sciences*, August, 1890,) after a careful study of the action of salol in acute tonsillitis and pharyngitis, adopts the summary of Gouguenheim as a proper guide for its administration :

1. Salol acts beneficially in acute anginas of whatever cause.
2. It quiets the pain and dysphagia with the greatest rapidity.
3. In quieting the pain it may shorten the duration of the quinsy.
4. It lowers the temperature.
5. In nearly all cases it diminishes the duration of the angina.
6. In order to attain those results, the dose should not be less than four grammes (sixty grains) daily.

